# Supplementary material for: Low Antibody-Dependent Enhancement of Viral Entry Activity Supports the Safety of Inactivated SARS-CoV-2 Vaccines
Source: Vaccines (Basel). 2025 Apr 18;13(4):425. doi: 10.3390/vaccines13040425 (PMC12031465; doi:10.3390/vaccines13040425)
Supplement: Supplementary file 1 [file vaccines-13-00425-s001.zip › Supplementary_Material.pdf]

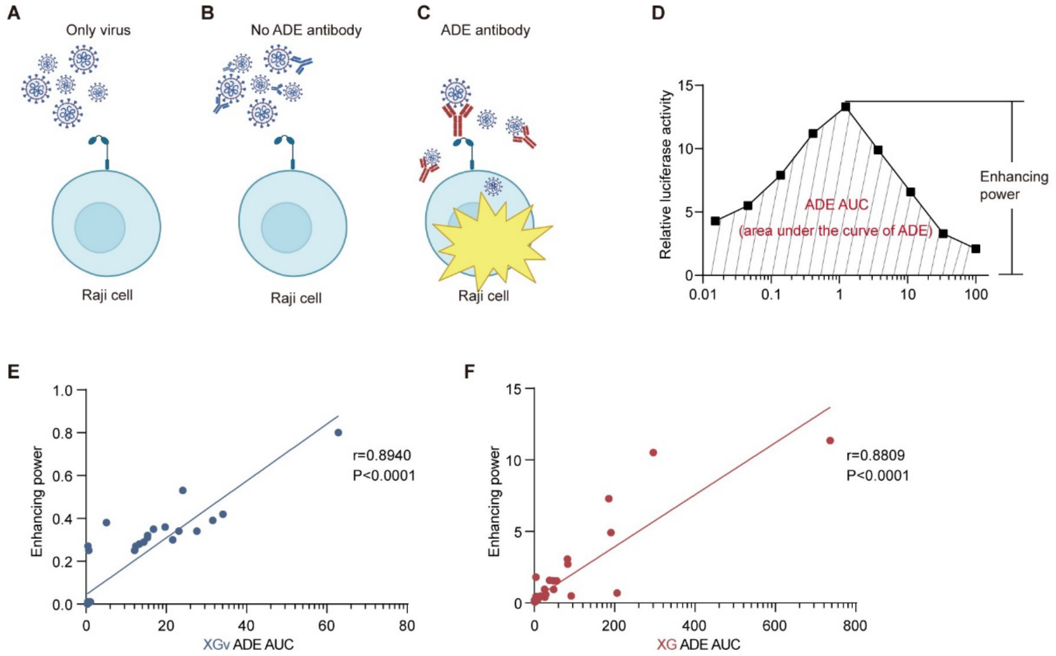

Figure S1. Schematic diagram for in vitro ADE assay in Raji cells. (A-C) Schematic diagram of the ADE assay in Raji cells in vitro. (A) Raji cells could not be infected by SARS-CoV-2 virus, since Raji cells have no expression of ACE2 receptor (A). Antibodies without ADE activity could also not induce SARS-CoV-2 viral entry into Raji cells (B). However, Raji cells have FcγRII (CD32) expression on the cell surface, and the ADE-inducing antibodies might facilitate ADE of viral entry through the Fc-FcγRII interaction (C). **(D)** Diagram of ADE area under the curve (AUC) and ADE enhancing power. **(E-F)** Correlation between ADE enhancing power and ADE AUC for XGv (E) and XG mAbs (F)

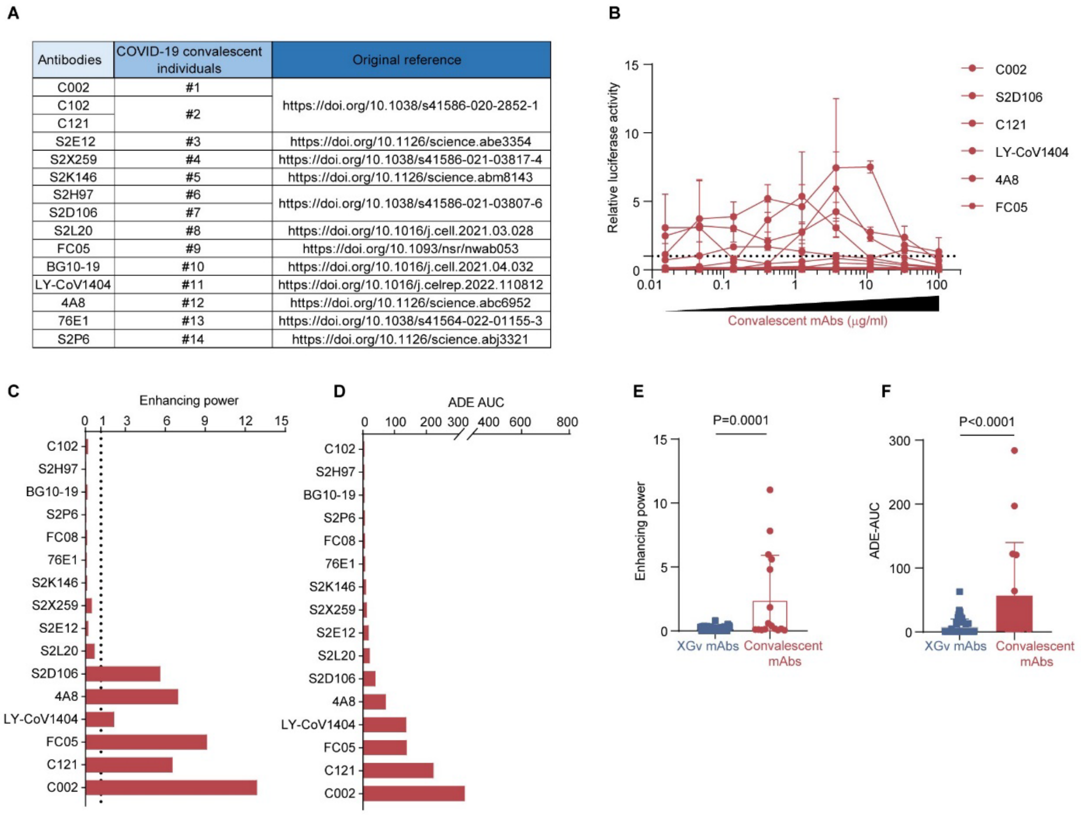

Antibodies	COVID-19 convalescent individuals	Original reference
C002	#1	https://doi.org/10.1038/s41586-020-2852-1
C102	#2	
C121		
S2E12	#3	https://doi.org/10.1126/science.abe3354
S2X259	#4	https://doi.org/10.1038/s41586-021-03817-4
S2K146	#5	https://doi.org/10.1126/science.abm8143
S2H97	#6	https://doi.org/10.1038/s41586-021-03807-6
S2D106	#7	
S2L20	#8	https://doi.org/10.1016/j.cell.2021.03.028
FC05	#9	https://doi.org/10.1093/nsr/nwab053
BG10-19	#10	https://doi.org/10.1016/j.cell.2021.04.032
LY-CoV1404	#11	https://doi.org/10.1016/j.celrep.2022.110812
4A8	#12	https://doi.org/10.1126/science.abc6952
76E1	#13	https://doi.org/10.1038/s41564-022-01155-3
S2P6	#14	https://doi.org/10.1126/science.abj3321

Figure S2. In vitro ADE activity of 15 convalescent mAbs. (A) List of the selected 15 previously published mAbs isolated from 14 convalescent donors. **(B)** In vitro ADE assays. Relative luciferase activity (y-axis) indicates the SARS-CoV-2 pseudoviral entry level induced by various concentrations (x-axis) of 15 convalescent mAbs. An ADE antibody, XG043 (2 μg/ml) was used as a reference for normalization, and its luciferase reading value was set as 1 and used as a cutoff for the ADE curves (dotted line). **(C)** ADE enhancing power of convalescent mAbs. (**D)** ADE AUC values of convalescent mAbs. **(E-F)** Statistical comparison of enhancing power (E) and ADE AUC (F) between XGv and 15 convalescent antibodies. The p values were calculated using the Mann-Whitney test.

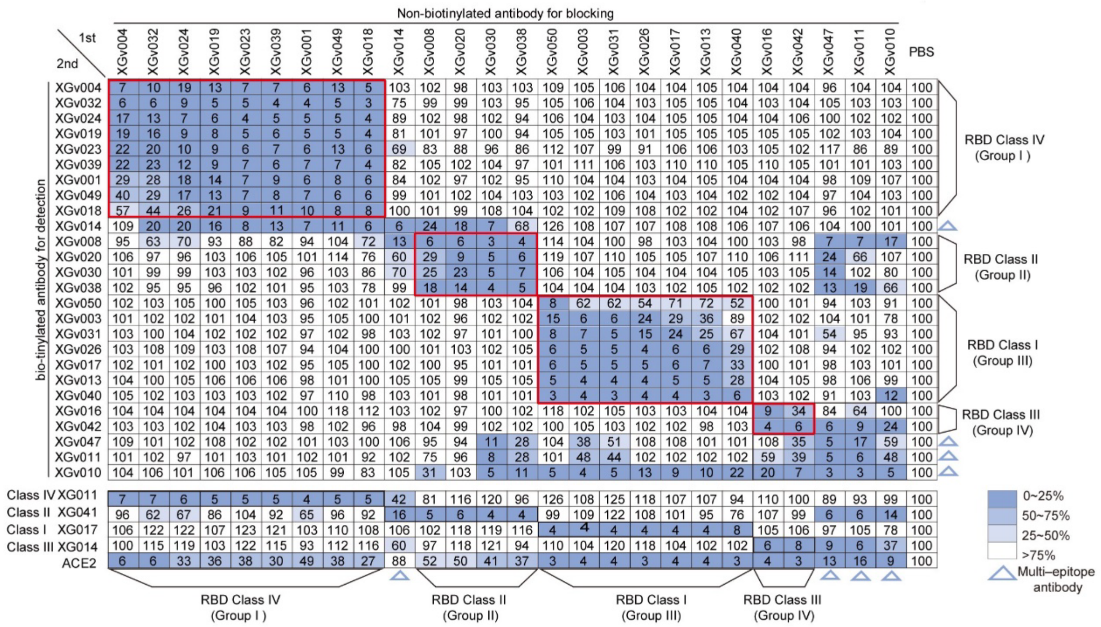

Figure S3. Competition ELISAs of RBD-binding XGv mAbs. Competition ELISAs to map the epitopes of RBD-binding XGv antibodies. Four non-overlapping RBD epitopes include RBD Class I, II, III, and VI (red rectangles). The antibodies targeting multiple overlapping RBD epitopes, XGv014, XGv047, XGv011, and XGv010, are indicated by blue triangles. The 1st unbiotinylated antibody (x-axis) is used for blocking RBD epitopes, while the 2nd biotinylated antibody (y-axis) is used for detection by using streptavidin-HRP. PBS was used as a negative control and a reference for normalization. All the tested antibodies blocked the binding of their biotinylated versions. XG011, XG041, XG017, and XG014 were used as control 2nd detection antibodies for RBD Class IV, II, I, and III, respectively. ACE2 was used as the 2nd detection antibody to determine the competitive capacity with ACE2.

Non-biotinylated antibody for blocking

2nd \ 1st	XGv002	XGv006	XGv026	XGv028	XGv034	XGv025	XGv036	XGv045	XGv015	XGv022	XGv033	XGv007	XGv044	XGv027	XGv046	XGv005	XGv043	FC05	PBS
XGv002	5	6	5	4	5	4	5	5	76	109	7	103	151	64	120	87	137	129	100
XGv006	26	5	21	10	6	3	3	3	101	96	105	99	104	101	104	105	109	110	100
XGv026	18	23	10	6	11	6	12	11	95	104	152	106	209	62	147	90	167	166	100
XGv028	79	86	38	4	3	8	5	3	105	104	99	104	104	104	103	105	105	104	100
XGv034	98	103	76	14	5	27	19	3	106	105	94	105	105	105	105	99	107	107	100
XGv025	97	102	102	103	103	8	3	3	103	104	98	103	101	103	104	102	104	104	100
XGv036	96	103	102	104	103	58	9	5	103	104	97	103	103	103	103	104	104	103	100
XGv045	101	106	105	107	103	99	46	15	108	106	99	106	100	106	104	106	103	103	100
XGv015	81	76	94	103	110	80	96	87	6	35	28	60	15	86	94	104	124	115	100
XGv022	119	61	97	102	166	145	70	150	11	7	11	5	8	87	89	99	122	97	100
XGv033	94	102	103	102	16	101	103	103	87	54	4	13	7	103	105	103	101	101	100
XGv007	95	103	102	100	104	101	101	102	103	103	94	9	5	102	103	104	102	103	100
XGv044	98	106	103	104	103	101	104	102	104	104	97	104	16	104	104	105	102	103	100
XGv027	94	102	101	100	121	74	138	110	80	64	77	15	11	4	6	28	154	141	100
XGv046	97	102	102	104	100	95	102	97	98	94	73	22	9	8	4	44	105	93	100
XGv005	97	100	103	102	81	101	104	87	97	97	37	77	104	7	5	4	106	104	100
XGv043	99	99	104	104	104	104	98	105	103	102	98	103	105	102	99	104	8	84	100
FC05	98	105	104	103	104	105	103	102	104	105	95	102	100	104	103	105	3	3	100
XG007	99	106	104	105	104	40	13	4	104	104	99	103	104	105	104	103	104	103	100
XG033	112	114	111	114	111	109	109	116	42	21	4	5	5	113	114	105	110	86	100
XG027	95	103	103	101	105	102	104	103	103	105	95	101	105	103	105	104	8	51	100

Rows: bio-tinylated antibody for detection. Columns XGv002–XGv045: NTD Group I; XGv015–XGv005: NTD Group II; XGv043, FC05: NTD Group III. Rows XGv002–XGv045: NTD Group I; XGv015–XGv005: NTD Group II; XGv043, FC05: NTD Group III.

0~25% | 50~75% | 25~50% | >75%

Figure S4. Competition ELISAs of NTD-binding XGv mAbs. Competition ELISAs to map the epitopes of NTD-binding XGv antibodies. Three groups of non-overlapping epitopes include NTD Group I, II, and III (red rectangles). All the tested antibodies blocked the binding of their biotinylated versions. XG007, XG033, and XG027 were used as control 2nd detection antibodies for NTD Group I, II, and III, respectively. Only one antibody, XGv043, was identified as an NTD Group III antibody, sharing the similar NTD epitope as FC05 antibody[1].

Reference

1. Zhang, L.; Cao, L.; Gao, X.S.; Zheng, B.Y.; Deng, Y.Q.; Li, J.X.; Feng, R.; Bian, Q.; Guo, X.L.; Wang, N.; et al. A proof of concept for neutralizing antibody-guided vaccine design against SARS-CoV-2. *Natl Sci Rev* **2021**, *8*, nwab053, doi:10.1093/nsr/nwab053.
